# Editorial Note: Mathematical modeling of the molecular switch of TNFR1-mediated signaling pathways applying Petri net formalism and *in silico* knockout analysis

**DOI:** 10.1371/journal.pcbi.1013003

**Published:** 2025-04-22

**Authors:** The PLOS Computational Biology Editors

After publication of this article [1], concerns were raised about the replicability of the results based on the descriptions provided in [1] and the publicly available software. Specifically:

- There appears to be a layout discrepancy between the model as visualized in Fig 2 in [1] and the model file provided in the Supporting Information (S1 Data) in SBML format in [1].
- Whether the transition, place invariants, and Manatee invariants central to the results in [1] can be obtained using the Petri net and MonaLisa software [2] referred to in the Methods section of [1].
- Whether the results of the knockout analysis presented in [1] can be reproduced using the Petri net and isiKnock software referred to in the Methods section of [1].

The corresponding author stated that since the publication of [1], they have developed a more efficient layout algorithm, which can result in a slightly changed layout of the Petri net model although the underlying graph structure remains the same. They therefore provided the SBML files with the analysis and layout of Fig 2 in [1] in S10, S11 Files. They also stated that all results are based on the invariant analysis without performing any simulation, and as the MIs represent vectors of transitions, they checked the different outcomes based on the transitions known to belong to survival, apoptosis, or necroptosis. The corresponding author provided S1 and S9 Files to support the results in [1], including the Petri net reactions (S1 and S5 Files).

A member of the *PLOS Computational Biology* Board stated that loading the SBML file (S1 Data in [1]) into MonaLisa [2] and using the “Show Petri net” function in the GUI to display a network visualization shows the elements of the Petri net described in [1], including the 118 places (species) and 130 transitions (reactions), which appear to correspond directly to those shown in Fig 2 in [1]. They also verified that the MonaLisa program [2] could be run with the MonaLisa.jar file available on GitHub [3] to reproduce the results reported in [1]; however, they noted that the analyses appear to run correctly in Windows only.

The corresponding author stated that using Linux Fedora on Lenovo and Unix workstations, the TNR1 model gives the same results as using Windows.

The *PLOS Computational Biology* Editors issue this Editorial Note to provide the above information to readers and the conditions for replicating the results in [1].

## Supporting information

S1 File The Petri net graph for the TNFR1 model in S2 Table, S3 Table and S1 Data in [1]. (TXT)

S2 File The transition invariants for the TNFR1 model in S5 Table in [1]. (INV)

S3 File The place invariants for the TNFR1 model in Table 1 in [1]. (INV)

S4 File The manatee invariants for the TNFR1 model in S6 Table in [1]. (INV)

S5 File The Petri net graph for the Salmonella model in S1 and S2 Tables in [1]. (TXT)

S6 File The in-silico knockout transition invariants for the Salmonella model in S3 Table in [1]. (INV)

S7 File The in-silico knockout place invariants for the Salmonella model in S4 Table in [1]. (INV)

S8 File Results of the isiKnock analysis. (ZIP)

S9 File Results of the TNFR1 analysis. (ZIP)

S10 File SBML file for the analysis of Fig 2 in [1]. (XML)

S11 File SBML file with the layout of Fig 2 in [1]. (XML)
