# Supplementary material for: Editorial Note: Mathematical modeling of the molecular switch of TNFR1-mediated signaling pathways applying Petri net formalism and in silico knockout analysis
Source: PLoS Comput Biol. 2025 Apr 22;21(4):e1013003. doi: 10.1371/journal.pcbi.1013003 (PMC12013866; doi:10.1371/journal.pcbi.1013003)
Supplement: S9 File — (ZIP) [file pcbi.1013003.s009.zip › TNFR1/TNFR1_plos.txt.pdf]

```
Syn_TNFR1:  -> TNFR1
Syn_TNF:  -> TNF
Syn_TRADD:  -> TRADD
Syn_RIP1:  -> RIP1
Syn_TRAF2:  -> TRAF2
Syn_clap:  -> clap
Syn_TAB:  -> TAB
Syn_TAK1:  -> TAK1
Syn_NEMO:  -> Nemo
Syn_IKK:  -> IKK
Syn_LUBAC:  -> LUBAC
Syn_CYLD:  -> CYLD
Syn_NF-kB:  -> NF-kB
Syn_FADD:  -> FADD
Syn_Procasp8:  -> Procasp8
Syn_SCF:  -> SCF
Syn_cFLIPs:  -> cFLIPs
Syn_RIP3:  -> RIP3
Syn_MLKL:  -> MLKL
Syn_Procasp3:  -> Procasp3
Syn_Bid:  -> Bid
Syn_BAX:  -> BAX
Syn_SMAC:  -> SMAC_mito
Syn_Cyt_c:  -> Cyt_c_mito
Syn_Apaf1:  -> Apaf1
Syn_Procasp9:  -> Procasp9
T1: TNFR1 + TNF -> TNFR1:TNF
T2: TRADD + TNFR1:TNF -> T:T:TRADD
T3: RIP1 + T:T:TRADD -> T:T:T:RIP
T4: TRAF2 + T:T:T:RIP -> T:T:T:R:TRAF2
T5: clap + T:T:T:R:TRAF2 -> T:T:T:R:T:cIAP
T6: T:T:T:R:T:cIAP -> T:T:T:R:T:c_K63ub
T42: T:T:TRADD -> T:T_diss + TRADD_c
T48: T:T:T:RIP -> T:T_diss + TRADD:RIP1
diss2: T:T_diss ->
T49: TRADD:RIP1 -> RIP_diss + TRADD_c
T43: TRADD_c + FADD -> T:FADD
T44: Procasp8 + T:FADD -> CIIa
deg1: RIP_diss ->
T50: TRADD:RIP1 -> RIP1_c + TRADD_diss
deg2: TRADD_diss ->
T53: RIP1_c + FADD -> R:FADD
T54: Procasp8 + R:FADD -> CIIb
T45: Procasp8 + CIIa -> CIIa:Pc8_2
T47: CIIa + cFLIPL -> CIIa:cFLIPL
Clla_inhib: CIIa:cFLIPL -> CIIa_diss
T46: CIIa:Pc8_2 -> CIIa_diss + CASP8
diss3: CIIa_diss ->
T59: cFLIPL + CIIb -> CIIb:cFLIPL
T60: CIIb:cFLIPL + RIP3 -> RIP1:RIP3_cl + Pc8:cFLIPL + CIIb_diss
T62: CIIb:Pc8_2 -> CIIb_diss + CASP8
diss5: CIIb_diss ->
Pc8_inhib: Pc8:cFLIPL ->
RIP1:RIP3_inhib: RIP1:RIP3_cl ->
T51: RIP1_c + RIP3 -> RIP1:RIP3
T56: RIP3 + CIIb:cFLIPs -> CIIb:cFs:RIP3
T55: cFLIPs + CIIb -> CIIb:cFLIPs
T57: CIIb:cFs:RIP3 + MLKL -> CIIb_diss + MLKL_p
T58: MLKL_p -> MLKL_PM
Necroptosis: MLKL_PM ->
T52: RIP1:RIP3 + MLKL -> R:R_diss + MLKL_p
diss4: R:R_diss ->
T61: Procasp8 + CIIb -> CIIb:Pc8_2
T7: TAB + T:T:T:R:T:c_K63ub -> K63ub:TAB
T8: K63ub:TAB -> TAB_K63ub + T:T:T:R:T:c_K63ub
T9: TAK1 + TAB_K63ub -> TAB:TAK_K63ub
T13: T:T:T:R:T:c_K63ub + TAB:TAK_K63ub + NEMO:IKK_K63ub + NEMO:IKK_M1ub -> CI
T12: IKK + NEMO_K63ub -> NEMO:IKK_K63ub
T11: K63ub:NEMO -> NEMO_K63ub + T:T:T:R:T:c_K63ub
T10: Nemo + T:T:T:R:T:c_K63ub -> K63ub:NEMO
T14: LUBAC + T:T:T:R:T:c_K63ub -> K63ub:LUBAC
```

```
T18: CYLD + K63ub:LUBAC -> K63ub:L:CYLD
T15: Nemo + K63ub:LUBAC -> K36ub:LUBAC:M1ub:NEMO
T16: K36ub:LUBAC:M1ub:NEMO -> NEMO_M1ub + T:T:T:R:T:c_K63ub
T17: IKK + NEMO_M1ub -> NEMO:IKK_M1ub
T22: CI + NF-kB:IkB -> CI:N:I
T37: NF-kB + IkB -> NF-kB:IkB
T40: NF-kB:IkB_n -> NF-kB:IkB
T39: IkB_n + NF-kB_n2 -> NF-kB:IkB_n
T38: IkB -> IkB_n
deg4: NF-kB ->
T23: CI:N:I -> NF-kB + CI + IkB_p
T25: NF-kB -> NF-kB_n
Syn_IkB: IkB_mRNA -> IkB
T26: NF-kB_n + IkB_g -> NF-kB_n:IkB_g
T27: NF-kB_n:IkB_g -> IkB_g + IkB_mRNA + NF-kB_n
T19: K63ub:L:CYLD -> LUBAC_K63ub:CYLD + K63ub:LUBAC
T20: CI + LUBAC_K63ub:CYLD -> CI:CYLD
T21: CI:CYLD -> TRADD:RIP1 + CI_diss
diss1: CI_diss ->
T41: CI + A20 -> CI:A20
T24: SCF + IkB_p -> IkB_K48ub
deg7: IkB_K48ub ->
T30: NF-kB_n -> NF-kB_n2 + NF-kB_n2
T28: NF-kB_n + A20_g -> NF-kB_n:A20_g
T29: NF-kB_n:A20_g -> A20_mRNA + NF-kB_n + A20_g
Syn_A20: A20_mRNA -> A20
T31: NF-kB_n2 + XIAP_g -> NF-kB_n2:XIAP_g
T32: NF-kB_n2:XIAP_g -> NF-kB_n2 + XIAP_g + XIAP_mRNA
Syn_XIAP: XIAP_mRNA -> XIAP
T33: NF-kB_n2 + cFLIP_g -> NF-kB_n2:cFLIP_g
T34: NF-kB_n2:cFLIP_g -> NF-kB_n2 + cFLIP_g + cFLIPL_mRNA
Syn_cFLIPL: cFLIPL_mRNA -> cFLIPL
T35: NF-kB_n2 + BCL-2_g -> NF-kB_n2:BCL-2_g
T36: NF-kB_n2:BCL-2_g -> NF-kB_n2 + BCL-2_g + BCL-2_mRNA
Syn_BCL-2: BCL-2_mRNA -> BCL-2
T63: Procasp3 + CASP8 -> CASP3 + CASP8_diss
Apoptosis: CASP3 ->
T64: XIAP + XIAP + CASP3 -> XIAP:CASP3
CASP3_inhib: XIAP:CASP3 ->
T65: Bid + CASP8 -> CASP8_diss + tBid
deg3: CASP8_diss ->
T66: tBid -> tBid_MOM
T67: BAX + tBid_MOM -> tBid:BAX
T69: BAX + tBid:BAX -> tBid:BAX_2
T70: tBid:BAX_2 -> BAX:BAX_pore
T68: BCL-2 + tBid:BAX -> BCL-2:BAX
BAX_inhib: BCL-2:BAX ->
T71: SMAC_mito + Cyt_c_mito + BAX:BAX_pore -> SMAC + Cyt_c
deg5: SMAC ->
T76: SMAC + XIAP:Pc9 -> SMAC:XIAP:Pc9
Apo_XIAP_inhib: SMAC:XIAP:Pc9 ->
Pc9_inhib: XIAP:Pc9 ->
T75: XIAP + XIAP + Apoptosome -> XIAP:Pc9
T72: Procasp9 + Apaf1 + Cyt_c -> Apoptosome
deg6: CASP9_diss ->
T74: Procasp3 + CASP9 -> CASP9_diss + CASP3
T73: Procasp9 + Apoptosome -> CASP9 + Apoptosome_diss
diss6: Apoptosome_diss ->
CI_diss: CI:A20 ->
```
